# Supplementary material for: MetaRibo-Seq measures translation in microbiomes
Source: Nat Commun. 2020 Jun 29;11:3268. doi: 10.1038/s41467-020-17081-z (PMC7324362; doi:10.1038/s41467-020-17081-z)
Supplement: Supplementary file 10 — Supplementary Data 7 [file 41467_2020_17081_MOESM10_ESM.zip › File2/Confidence_High_Taxonomy/387899_out.krona.html]

Javascript must be enabled to view this page.

members
magnitude
magnitudeUnassigned
count
unassigned
taxon
rank

387899\_out

22

2
22
superkingdom

1239
22
phylum

1737404
3
class

1737405
3
order

1570339
3
family

3
165779
genus

54007
3

SRS020263\_contig\_number\_1258SRS058182\_contig\_number\_contig-100\_592.16563SRS100036\_contig\_number\_1157
species

class
19
186801

19

SRS055017\_contig\_number\_contig-100\_1516.209914
order
186802
1

family

SRS056519\_contig\_number\_19107
13
541000
1

genus
1263
5

species

SRS055982\_contig\_number\_19064SRS104311\_contig\_number\_17389
2
2293244

species

SRS017916\_contig\_number\_contig-100\_577.121465SRS018351\_contig\_number\_2546SRS052078\_contig\_number\_contig-100\_1197.41787
2293167
3

946234
1
genus

292800
1
species

SRS077454\_contig\_number\_8169

genus
216851
6


SRS015960\_contig\_number\_contig-100\_1425.141705SRS054956\_contig\_number\_11661SRS104197\_contig\_number\_contig-100\_281.6440SRS143598\_contig\_number\_14453SRS146888\_contig\_number\_contig-100\_1311.94053SRS147271\_contig\_number\_contig-100\_2041.210674
species
853
6

family
3
186803

genus
1
33042

species

SRS014613\_contig\_number\_19370
116085
1

genus
1506553
2


SRS017521\_contig\_number\_48164
species
1
1531

1
460384
species

SRS148159\_contig\_number\_7130

31979
2
family

genus
580596
1

2292296
1

SRS149879\_contig\_number\_contig-100\_238.163929
species

genus
1
1649459

154046
1

SRS143070\_contig\_number\_17663
species
